# The Potential Application of Resveratrol and Its Derivatives in Central Nervous System Tumors

**DOI:** 10.3390/ijms252413338

**Published:** 2024-12-12

**Authors:** Agnieszka Nowacka, Martyna Śniegocka, Wojciech Smuczyński, Sara Liss, Ewa Ziółkowska, Dominika Bożiłow, Maciej Śniegocki, Michał Wiciński

**Affiliations:** 1 Department of Neurosurgery, Nicolas Copernicus University in Toruń, Collegium Medicum in Bydgoszcz, ul. Curie Skłodowskiej 9, 85-094 Bydgoszcz, Poland; sniegocki@cm.umk.pl; 2 Department of Anatomical, Histological, Forensic & Orthopedic Sciences, Section of Histology & Medical Embryology, Sapienza University of Rome, Via A. Scarpa, 14-16, 00161 Rome, Italy; martyna.sniegocka@gmail.com; 3 Department of Physiotherapy, Nicolas Copernicus University in Toruń, Collegium Medicum in Bydgoszcz, ul. Techników 3, 85-801 Bydgoszcz, Poland; w.smuczynski@cm.umk.pl; 4 Department of Pharmacology and Therapeutics, Nicolas Copernicus University in Toruń, Collegium Medicum in Bydgoszcz, ul. Curie Skłodowskiej 9, 85-090 Bydgoszcz, Poland; sara-liss@wp.pl (S.L.); michal.wicinski@cm.umk.pl (M.W.); 5 Department of Pediatrics, Washington University School of Medicine, St. Louis, MO 63110, USA; eziolkowska@wustl.edu; 6 Anaesthesiology and Intensive Care Clinical Ward, The 10th Military Research Hospital and Polyclinic, ul. Powstańców Warszawy 5, 85-681 Bydgoszcz, Poland; bozilow@wp.pl

**Keywords:** resveratrol, RVS, glioma, glioblastoma, meningioma, medulloblastoma, CNS tumors, central nervous system tumors, pterostilbene, PTE

## Abstract

Resveratrol, a naturally occurring polyphenolic compound found in various plants, has been extensively studied for its broad spectrum of beneficial biological effects. These encompass its potent antioxidant properties, anti-inflammatory activities, anti-aging capabilities, cardioprotective functions, and neuroprotective potential. The diverse biological actions of resveratrol extend beyond these well-established properties. It also exerts a significant impact on metabolic processes and bioavailability, and critically, it demonstrates the ability to effectively traverse the blood–brain barrier. This capacity to penetrate the central nervous system renders resveratrol a promising therapeutic agent for the management of central nervous system malignancies, as it has been shown to inhibit tumor cell proliferation, induce apoptosis, and modulate key signaling cascades, such as PI3K/Akt, JAK/STAT, and NF-kB. The multifaceted nature of resveratrol’s biological effects, including its influence on diverse physiological processes, underscores its potential as a valuable therapeutic option for the treatment of central nervous system tumors.

## 1. Resveratrol—An Introduction and Historical Overview

Resveratrol (RVS), a naturally occurring polyphenol and phytoalexin, is predominantly found in various plant-based foods, particularly in the skins and seeds of grapes, as well as in several berries and nuts [1,2,3,4,5,6,7]. Phytoalexins are specialized plant metabolites produced in response to stressors such as injury, UV (ultraviotel) radiation, fungal infection, or other pathogenic attacks [8,9]. The biosynthesis of resveratrol in plants follows the phenylalanine pathway, initially producing the trans-resveratrol form [8]. This form can then isomerize to cis-resveratrol or be glycosylated to form trans- and cis-piceid by resveratrol 3-O-beta-glycosyltransferases [10,11,12,13]. The stilbene synthesis pathway, responsible for resveratrol production, branches off from the phenylpropanoid pathway and is considered an extension of the flavonoid pathway. It is characterized by the action of specific enzymes and transcription factors that regulate the biosynthesis of stilbenes, particularly in grapevines [14,15,16,17,18].

The initial identification of resveratrol dates back to 1939 when Michio Takaoka isolated it from the roots of Veratrum grandiflorum O. Loes [19]. Later, in 1963, resveratrol was identified as a constituent of Polygonum cuspidatum [20]. A method for detecting trans-resveratrol was reported in 1976 [21]. However, significant interest in resveratrol surged after 1992, following Renaud and de Lorgeril’s description of the “French paradox” [22,23]. This paradox refers to the observation of a relatively low incidence of coronary heart disease in French people despite their consumption of a diet rich in cholesterol and saturated fat [24,25,26,27]. This phenomenon sparked curiosity and led to investigations into potential protective factors within the French diet and lifestyle.

**Figure 1:**
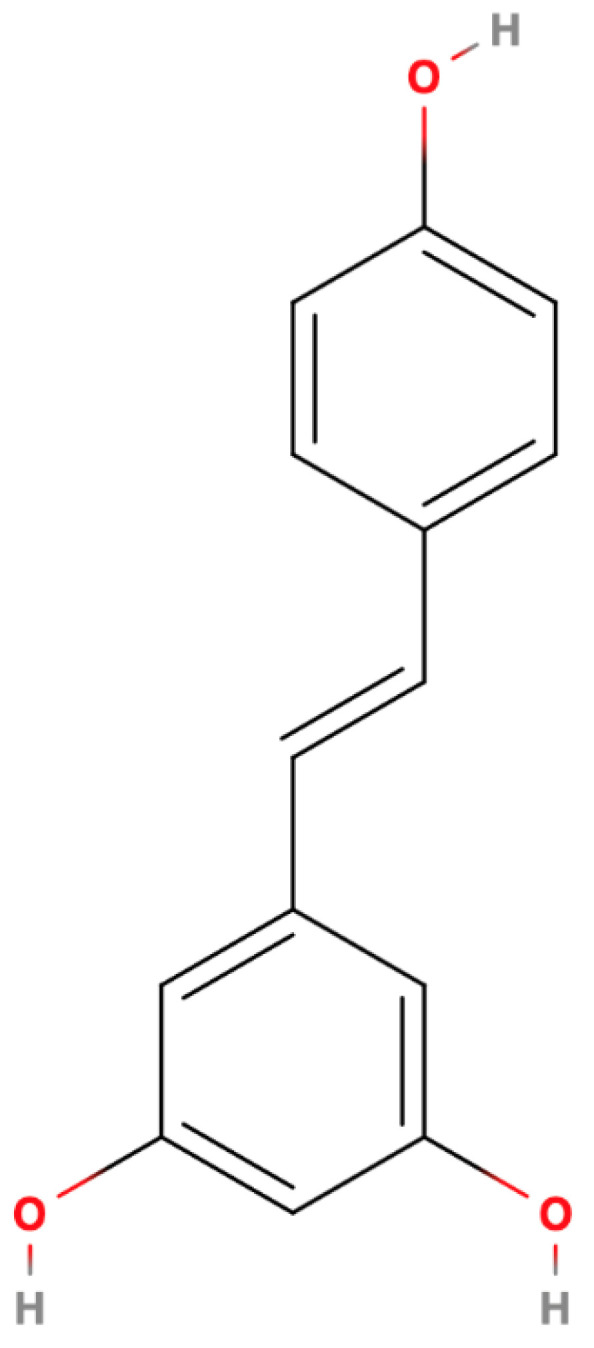
Resveratrol—molecular structure.

Resveratrol (3,5,4′-trihydroxy-trans-stilbene) is a stilbenoid, with a molecular weight of 228.2 g/mol and a melting point of 253–255 °C [7,28,29,30,31]. Its chemical structure consists of two aromatic rings (phenol groups) linked by a styrene double bond [30,31,32] (Figure 1).

Phenolic groups, characterized by a hydroxyl (-OH) group bonded to an aromatic ring, are essential structural components of resveratrol and contribute significantly to its biological activity [7]. Resveratrol possesses two phenolic rings, each bearing hydroxyl groups that are key to its antioxidant and anti-inflammatory properties [30,32]. These hydroxyl groups act as hydrogen atom donors, enabling RVS to effectively neutralize free radicals and protect cells from oxidative damage [32]. The phenolic groups play a crucial role in resveratrol’s diverse biological activities, including its antioxidant effects, its ability to modulate inflammatory pathways, and its neuroprotective properties [32]. The specific arrangement and presence of these phenolic groups are critical for the structure–activity relationship of resveratrol, and modifications to these groups can significantly influence its efficacy and stability [32]. Consequently, research on resveratrol derivatives often focuses on optimizing these functional groups to enhance bioavailability and pharmacological activity, aiming to develop more effective resveratrol-based clinical drugs.

**Figure 2:**
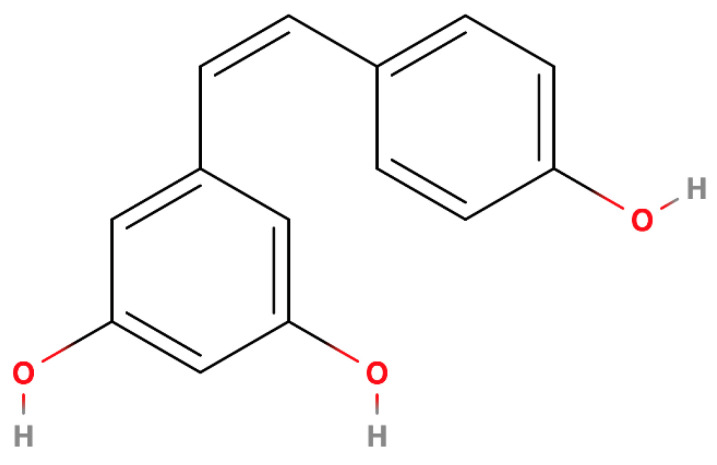
Cis-resveratrol—molecular structure.

Resveratrol exists as two primary isomers, *trans*-resveratrol and *cis*-resveratrol, distinguished by the spatial arrangement of functional groups around the molecule’s double bond [28,30,31,32,33]. *Trans*-resveratrol (Figure 1) is the more stable and biologically active form, exhibiting greater efficacy in terms of antioxidant, anti-inflammatory, and neuroprotective properties [7,28,31,32]. This enhanced activity is attributed to its favorable configuration for interacting with biological targets. *Cis*-resveratrol (Figure 2), while less common, can be generated from *trans*-resveratrol through exposure to light or heat [28,31,32]. However, its biological activity is less pronounced compared to the *trans* isomer.

The distinct properties of these isomers are crucial for understanding resveratrol’s medicinal applications, with *trans*-resveratrol being the primary focus of research and development for resveratrol-based therapies due to its stability and efficacy. Ongoing research explores structural modifications of resveratrol to enhance its bioavailability and stability, primarily focusing on the *trans* form, with the goal of developing synthetic analogs that retain its beneficial properties while addressing limitations associated with the natural compound.

Resveratrol contains three hydroxyl (-OH) groups, two on the *A* ring and one on the *B* ring of its stilbenoid structure [28,32]. These hydroxyl groups significantly influence their chemical properties and are essential for biological activities, influencing their solubility, reactivity, and interactions with biological targets [30,32]. The hydrogen-donating ability of these groups allows resveratrol to act as a potent antioxidant, neutralizing free radicals and protecting cells from oxidative damage [31,32]. Furthermore, the hydroxyl groups contribute to resveratrol’s anti-inflammatory effects by modulating inflammatory pathways [31,32]. The combined antioxidant and anti-inflammatory properties mediated by the hydroxyl groups also contribute to resveratrol’s neuroprotective potential. The number and arrangement of hydroxyl groups are critical for resveratrol’s structure-activity relationship, and modifications can significantly impact its efficacy and stability [32]. Therefore, understanding the role of hydroxyl groups is crucial for developing resveratrol derivatives with improved pharmacological activities and bioavailability for clinical applications.

Glycosylation of resveratrol can occur resulting in piceid, where a glucose moiety is attached to one of the hydroxyl groups [12,30,31,32]. It can significantly impact resveratrol’s properties and biological activity. Glycosylation can enhance resveratrol’s bioavailability and stability by increasing its solubility and prolonging its circulation time in the body, counteracting the limitations of natural resveratrol’s poor solubility and rapid metabolism [12,34]. Moreover, glycosylation can alter resveratrol’s biological activity, potentially enhancing its antioxidant and anti-inflammatory effects and overall pharmacological efficacy by influencing its interaction with biological targets [12,34]. Glycosylated resveratrol derivatives show promise for various therapeutic applications, including cancer treatment, cardiovascular health, and neuroprotection, as they may lead to more effective treatments for diseases involving oxidative stress and inflammation.

## 2. Metabolism

Resveratrol’s extensive metabolism significantly impacts its bioavailability and potential therapeutic effects. The rapid formation of metabolites contributes to its short half-life in the body. Understanding the metabolic pathways and the activity of the metabolites is crucial for developing effective resveratrol-based therapies.

**Figure 3:**
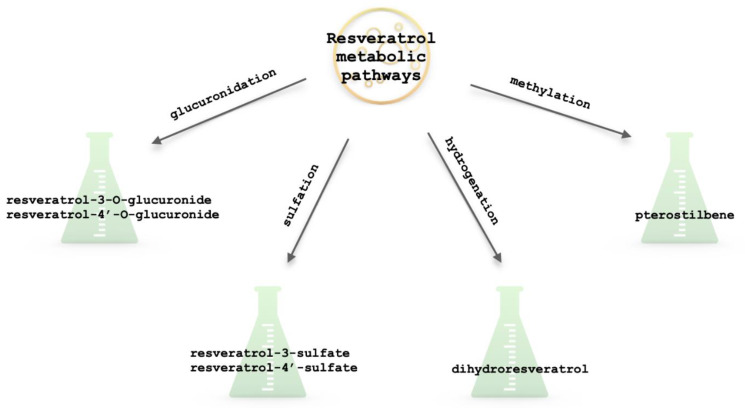
Resveratrol metabolic pathways.

Resveratrol is rapidly absorbed after ingestion, but its bioavailability is low due to extensive first-pass metabolism, primarily in the liver and intestines. While Phase I metabolism, involving oxidation and reduction reactions catalyzed by enzymes like cytochrome P450, does occur, the major metabolic pathways are Phase II conjugation reactions (Figure 3) [32,35,36,37]:

- Glucuronidation: This is the major metabolic pathway for resveratrol. It is conjugated with glucuronic acid by UDP-glucuronosyltransferases, primarily UGT1A1 and UGT1A9 [30,32,35,36,37,38]. This process forms resveratrol glucuronides, such as resveratrol-3-O-glucuronide and resveratrol-4′-O-glucuronide [30,32,35,38]. These glucuronides are more water-soluble and are readily excreted in urine and bile.
- Sulfation: Resveratrol can also be sulfated by sulfotransferases, mainly SULT1A1 and SULT1E1, to form resveratrol sulfates like resveratrol-3-sulfate and resveratrol-4′-sulfate [30,32,35,36,37,38,39]. These conjugated metabolites are more water-soluble, facilitating their excretion in urine and bile [35,39,40].

These Phase II metabolites are often found in higher concentrations in the body than the parent compound due to resveratrol’s rapid metabolism. The metabolites may possess different biological activities compared to resveratrol itself [35,38]. Some may retain or even have enhanced antioxidant and anti-inflammatory properties, while others may show reduced efficacy [30,35,38].

Resveratrol is also known to undergo alternative metabolic reactions (Figure 3), such as:

- Hydrogenation: Resveratrol can be hydrogenated in the gut by the microflora to form dihydroresveratrol, formed by the saturation of the double bond in the stilbene structure [33,36,41,42]. It has been shown to possess distinct biological activities compared to resveratrol—some studies suggest that dihydroresveratrol may have enhanced antioxidant properties and potentially different effects on cell signaling pathways [42].
- Methylation: Methylation involves the addition of a methyl group to the resveratrol molecule, primarily at hydroxyl groups, by methyltransferase enzymes using S-adenosylmethionine as the methyl donor [43]. A key example is the methylation of resveratrol to form pterostilbene, a dimethylated analog [43,44]. Methylation can increase resveratrol’s stability and absorption in the gastrointestinal tract, potentially leading to improved bioavailability. Methylated metabolites may exhibit different pharmacological properties compared to resveratrol, potentially enhancing antioxidant, anti-inflammatory, and even anti-cancer effects [36,43,44].

Resveratrol metabolism can be influenced by several factors such as [45,46]:

- Individual genetic variations in UGT (UDP-glucuronosyltransferase) and SULT (Sulfotransferase) enzyme activity can affect metabolism rates.
- Gut microbiome composition can modulate the production of hydrogenated resveratrol metabolites.
- Dosage and route of administration—with higher doses and different routes potentially altering the rate and extent of metabolic processes.
- The food matrix, including other compounds present in food, can influence both resveratrol absorption and its subsequent metabolism.

## 3. Bioavailability

Resveratrol exhibits high absorption (up to 75%) in humans following oral administration, primarily via transepithelial diffusion [30,47,48]. However, its extensive metabolism results in low systemic bioavailability (reduced to less than 1%) [28], with peak plasma concentrations typically in the range of 0.1–1 μM after oral administration of a single dose [47,48,49]. This low bioavailability poses a significant challenge to realizing resveratrol’s therapeutic potential, prompting research into methods for its enhancement. One of them is the use of alternative routes of administration, such as oral mucosal delivery, which have shown promise for bypassing first-pass metabolism and achieving higher bioavailability compared to oral ingestion [50]. The other successful strategy involves encapsulating resveratrol in casein nanoparticles, resulting in a tenfold increase, up to 26.5%, in oral bioavailability [51]. Other approaches include nanoencapsulation in lipid nanocarriers and liposomes, nanoemulsions, micelles, insertion into polymeric particles, solid dispersions, and nanocrystals [52,53]. Very promising for enhancing RVS bioavailability seems to be the use of a water-in-oil-in-water (W/O/W) emulsion system [54]. The emulsion improves resveratrol’s physicochemical stability and resistance to in vitro digestion, leading to increased absorption in the gastrointestinal tract [54]. Furthermore, the emulsion’s transport properties promote better uptake across intestinal barriers [54]. These findings suggest that W/O/W emulsions offer a promising delivery system for resveratrol, potentially leading to more effective nutraceutical applications.

Resveratrol’s therapeutic potential is significantly hampered by its low bioavailability, not only due to its extensive metabolism but also poor water solubility [55]. The limited water solubility restricts the amount of resveratrol that can be absorbed in the gastrointestinal tract, reducing the amount reaching the systemic circulation. Compounding this issue, resveratrol undergoes rapid and extensive metabolism in the liver, predominantly through glucuronidation. This process converts resveratrol into glucuronide conjugates, which are generally less active than the parent compound. Consequently, only a small fraction of orally administered RVS reaches its intended targets in the body. To overcome these limitations, a dual strategy involving the combination of resveratrol with glycyrrhetinic acid in a phospholipid complex has shown promise [55]. This RES/GA-PC (resveratrol with glycyrrhetinic acid in a phospholipid complex) formulation enhances resveratrol’s water solubility, facilitating improved absorption, and simultaneously inhibits glucuronidation, reducing its metabolic clearance [55]. The resulting increase in bioavailability is substantial, with a reported 2.49-fold increase in AUC0-10 and a 1.45-fold improvement in the proportion of absorbed resveratrol [55]. These findings underscore the importance of addressing both solubility and metabolism to maximize resveratrol’s therapeutic efficacy.

## 4. Biological Effects

**Figure 4:**
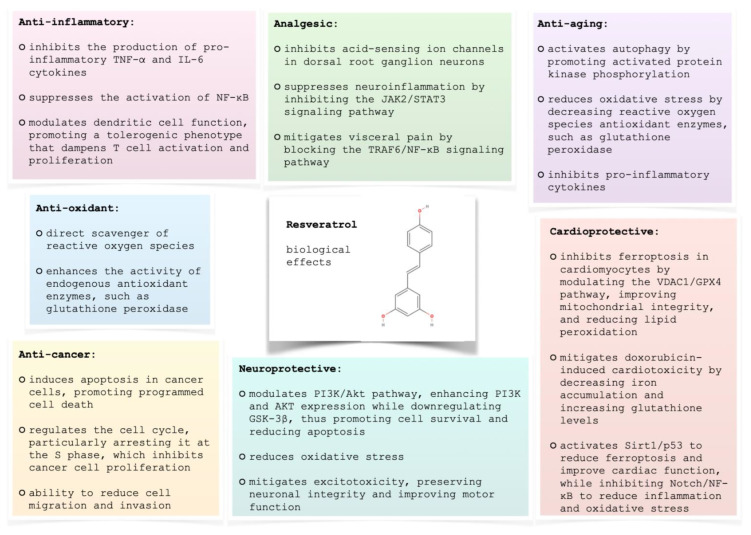
Resveratrol biological effects.

Resveratrol has been extensively studied for its wide range of biological effects (Figure 4) which include:

- Anti-inflammatory properties [4,56,57,58,59,60,61]: Resveratrol exerts its anti-inflammatory effects through multiple mechanisms. It inhibits the production of pro-inflammatory cytokines like TNF-α and IL-6, key players in the inflammatory cascade [57,62]. Additionally, resveratrol suppresses the activation of NF-κB, a crucial transcription factor regulating inflammatory responses [58]. Furthermore, it modulates dendritic cell function, promoting a tolerogenic phenotype that dampens T-cell activation and proliferation [62]. By targeting these diverse pathways, RVS effectively mitigates inflammation.
- Anti-oxidant properties [61,63,64,65,66,67,68]: Resveratrol’s antioxidant activity stems from a combination of direct and indirect mechanisms. It acts as a direct scavenger of reactive oxygen species, effectively neutralizing these harmful molecules [66]. Furthermore, RVS enhances the activity of endogenous antioxidant enzymes, such as glutathione peroxidase, bolstering the cellular defense system against oxidative stress [64,65]. This dual action makes resveratrol a valuable asset in managing oxidative stress.
- Anti-aging properties [69,70,71,72,73]: Resveratrol demonstrates several mechanisms that contribute to its potential anti-aging effects. It activates autophagy, a crucial cellular process for maintaining homeostasis and repair, by promoting AMP-activated protein kinase phosphorylation [69]. Resveratrol also reduces oxidative stress, a key factor in aging and cellular senescence, by decreasing reactive oxygen species [69,71]. Furthermore, it inhibits pro-inflammatory cytokines, offering protection against age-related diseases [72].
- Cardioprotective properties [68,72,74,75,76,77,78,79,80]: Resveratrol exhibits promising cardioprotective effects through multiple mechanisms. Studies show it inhibits ferroptosis, a form of regulated cell death, in cardiomyocytes by modulating the VDAC1/GPX4 (voltage-dependent anion channel 1 and glutathione peroxidase 4) pathway, improving mitochondrial integrity, and reducing lipid peroxidation [74]. It also mitigates doxorubicin-induced cardiotoxicity by decreasing iron accumulation and increasing glutathione levels [75]. Resveratrol modulates key signaling pathways, activating Sirt1/p53 to reduce ferroptosis and improve cardiac function [77], while inhibiting Notch/NF-κB to reduce inflammation and oxidative stress [72]. These mechanisms contribute to improved cardiac function and reduced infarct size in various experimental models, highlighting resveratrol’s therapeutic potential for cardiovascular diseases [76].
- Neuroprotective properties [81,82,83,84,85,86,87,88,89,90,91]: Resveratrol shows promise as a neuroprotective agent due to its multi-faceted mechanisms of action. It modulates critical signaling pathways, such as the PI3K/Akt pathway, enhancing PI3K and AKT expression while downregulating GSK-3β, which is crucial in Alzheimer’s disease models, thus promoting cell survival and reducing apoptosis [81]. RVS also reduces oxidative stress, as evidenced by lowered oxidative stress markers and improved antioxidant levels in ischemia-reperfusion injury models [82]. Furthermore, it mitigates excitotoxicity, preserving neuronal integrity and improving motor function in spinal cord injury models [84]. Novel delivery methods, such as intranasal administration of resveratrol nanoparticles, enhance bioavailability and neuroprotection, particularly in multiple sclerosis models, offering improved therapeutic outcomes [85].
- Analgesic properties [92,93,94,95,96,97,98]: Resveratrol demonstrates analgesic properties through several mechanisms. It inhibits acid-sensing ion channels in dorsal root ganglion neurons, reducing acid-induced pain [93]. Resveratrol also suppresses neuroinflammation by inhibiting the JAK2/STAT3 signaling pathway, thereby decreasing pro-inflammatory cytokines like TNF-α (tumor necrosis factor α) and IL-6 (interleukin 6) in spinal cord injury models, which contributes to alleviating mechanical allodynia [98]. Furthermore, it mitigates visceral pain by blocking the TRAF6/NF-κB signaling pathway, a key player in inflammatory pain [95].
- Anti-cancer properties [4,6,58,99,100,101,102,103,104,105,106,107,108,109]: Resveratrol holds promise as an anti-cancer agent due to its diverse mechanisms of action. It induces apoptosis in cancer cells, promoting programmed cell death [102]. Additionally, RVS regulates the cell cycle, particularly arresting it at the S phase, which inhibits cancer cell proliferation [6]. Its ability to reduce cell migration and invasion further limits cancer spread [6,103]. Clinical applications have shown resveratrol’s efficacy with minimal adverse effects, suggesting its potential as a chemotherapeutic agent [6]. Furthermore, nanoformulations are being explored to address its poor bioavailability.

## 5. Toxicity

Resveratrol demonstrates a remarkably low toxicity profile, supported by extensive research in both animal models and clinical settings. Studies have consistently shown minimal adverse effects, even at high doses, positioning resveratrol as a promising candidate for therapeutic applications. A key study by Johnson et al. involving rats and dogs established a No Observed Adverse Effect Level of 200 mg/kg/day for rats and 600 mg/kg/day for dogs, indicating minimal toxicity [110]. Further reinforcing its safety, Edwards et al. determined an acceptable daily intake of 450 mg/day for high-purity trans-resveratrol (resVida^®^), a level significantly exceeding typical dietary intake [111]. Beyond its inherent safety, resveratrol has also exhibited protective effects. Agarwal et al. reported its ability to reduce transplant-related toxicities in chemotherapy patients, suggesting its potential as an adjunct therapy to mitigate adverse effects [112]. Moreover, Radeva et al. demonstrated resveratrol’s cardioprotective properties when co-administered with doxorubicin, reducing the drug’s cardiotoxicity while simultaneously enhancing its efficacy against lymphoma cells [113]. While some studies have noted minor side effects at high doses, such as increased bilirubin levels in rats [110], these are generally considered insignificant and do not detract from resveratrol’s overall strong safety profile.

## 6. Resveratrol and the Blood–Brain Barrier

Resveratrol has shown promise as a neurological disorders therapeutic agent [114,115,116,117,118], but its limited blood–brain barrier (BBB) permeability poses a challenge. However, modified forms like resveratrol oligosaccharides demonstrate enhanced BBB penetration [119], potentially increasing their neuroprotective and cognitive benefits. These oligosaccharides, synthesized enzymatically, have shown improved spatial learning in mice, suggesting potential for cognitive enhancement and neuroprotection. Nanoparticles and lipid carriers further enhance resveratrol’s stability and absorption, facilitating BBB transport [115,120]. Another strategy enhancing BBB penetration is intranasal delivery with the use of nanocarriers, which bypasses hepatic metabolism and allows direct transport to the brain [117,121,122].

In addition to the expanding possibilities for overcoming the difficulties associated with blood–brain barrier penetration, there are also other bioavailability challenges that warrant further exploration. Recent studies showed that resveratrol’s extensive metabolism into glucuronides and sulfates limits its brain concentration [120], and glucose levels influence its absorption [123]. Further research and clinical validation are needed to optimize resveratrol’s therapeutic application in neurodegenerative diseases.

## 7. Routes of Administration in Brain Tumors

Resveratrol offers potential as an adjuvant therapy for brain tumors, especially glioblastoma, but its bioavailability remains a challenge. Conventional routes like oral administration and intraperitoneal injection result in low central nervous system concentrations [124]. External carotid artery injection delivers resveratrol directly to the tumor, achieving growth-inhibiting concentrations [125]. Direct intratumoral injection further maximizes local drug concentration, effectively suppressing tumor growth without systemic toxicity [38,100]. This method effectively achieves therapeutic doses at the tumor site [38,100]. Similarly, peritumoral injection, where RVS is administered around the tumor, also effectively inhibits tumor growth by utilizing the tumor’s vasculature for enhanced drug delivery [38,100]. Both methods offer targeted approaches for maximizing resveratrol’s anti-tumor effects. Intrathecal administration significantly increases brain RVS concentrations, enhancing anti-tumor effects and improving survival rates in preclinical models [124,126]. By directly administering resveratrol into the cerebrospinal fluid, lumbar punction (LP) injection achieves significantly higher brain concentrations compared to other routes [124]. The rapid attainment of peak brain concentration after LP injection demonstrates its efficacy in bypassing the blood–brain barrier. This targeted delivery results in markedly improved resveratrol bioavailability within glioblastoma tissues, suggesting its potential as a promising therapeutic strategy for brain malignancies. Despite these advancements, optimizing RVS delivery for improved clinical outcomes remains an area of ongoing research.

## 8. Resveratrol and Temozolomide

Resveratrol and temozolomide show promise as a synergistic combination therapy for glioblastoma [127], a highly aggressive brain tumor characterized by resistance to chemotherapy. This resistance is often driven by elevated levels of O6-methylguanine-DNA methyltransferase and activation of the STAT3 signaling pathway [128,129]. Resveratrol improves the chemosensitivity of GBM (glioblastoma) cells to TMZ (temozolomide) by downregulating STAT3 activity and its associated gene products, resulting in decreased cell proliferation and migration, and increased apoptosis [128,129]. Furthermore, RVS increases the expression of negative regulators of STAT3, such as PIAS3, SHP1, SHP2, and SOCS3, further amplifying its inhibitory effects [128,129]. The combination of resveratrol and TMZ not only reverses TMZ resistance but also decreases MGMT (O6-methylguanine-DNA methyltransferase) levels, a critical factor given MGMT’s role in counteracting TMZ’s effects [128,129]. Experimental evidence, including CCK-8 (cholecystokinin-8) assays and flow cytometry, demonstrates that the combination treatment leads to higher apoptosis rates compared to either treatment alone, highlighting resveratrol’s potential to potentiate TMZ’s anti-tumor activity [130]. These findings suggest that resveratrol may be a valuable addition to TMZ-based chemotherapy regimens for GBM.

Nanostructured lipid carriers offer a promising drug delivery system for this combination, significantly improving drug penetration and bioavailability compared to traditional formulations [131,132]. Studies by Mittal et. al. demonstrate the potential of engineered lactoferrin-conjugated nanostructured lipid carriers for enhanced glioblastoma treatment. The optimized LTR-NLC (Long-Chain Triglyceride Nanostructured Lipid Carriers) formulation effectively co-delivers temozolomide and resveratrol, achieving desirable characteristics like a particle size of 209.3 nm, high transmittance, and excellent drug entrapment efficiency [131,132]. Critically, LTR-NLC significantly improved drug penetration into the brain, showing a nearly threefold increase compared to traditional drug suspensions [131,132]. The synergistic activity of temozolomide and resveratrol within this delivery system further enhances therapeutic efficacy, as evidenced by improved IC50 values compared to drug suspension alone [131,132]. These findings strongly support the further development of LTR-NLC as a promising strategy for improving glioblastoma treatment outcomes.

## 9. Radiosensitizing Agent

Resveratrol is showing promise as a radiosensitizing agent, enhancing radiotherapy’s efficacy across various cancer types. It operates through multiple mechanisms:

- It amplifies radiation-induced DNA damage, leading to increased apoptosis in cancer cells [133,134,135], as evidenced in glioblastoma [133] and breast cancer models [136];
- It suppresses store-operated calcium entry (SOCE) by downregulating STIM1 and Orai1, further contributing to cell death under irradiation [137];
- It promotes reactive oxygen species (ROS) accumulation by reducing antioxidant enzyme activity, also promoting apoptosis [136].

Beyond direct effects on tumor cells, resveratrol enhances antitumor immunity by increasing CD8+ T cell populations and immune-related protein expression [133]. Interestingly, it displays a dual effect on normal tissues, acting as both a protector and sensitizer depending on the dosage, which suggests a complex role in radiotherapy [138]. While preclinical findings are promising, further research is needed to translate these effects into clinical applications and confirm resveratrol’s efficacy and safety in humans [139,140].

Lu et al. successfully isolated and characterized cancer stem-like cells from medulloblastoma (MB) tissues using a specific culture medium [141]. These MB-CSCs (Medulloblastoma-Cancer Stem-like Cells) exhibited key stem-like characteristics, including the formation of spheroid structures, high self-renewal capacity, and significant expression of stem cell markers such as CD133, Oct-4, Nanog, Nestin, and Musashi-1. Importantly, these MB-CSCs demonstrated a notable resistance to radiotherapy, a common challenge in cancer treatment, attributed to the inherent resilience of CSCs. However, treatment with 100 μM resveratrol significantly inhibited MB-CSC proliferation and enhanced their radiosensitivity. RVS treatment alone reduced MB-CSC viability by 40–45%, demonstrating a strong cytotoxic effect. Furthermore, the combination of RVS and ionizing radiation resulted in a synergistic effect, markedly decreasing MB-CSC viability and inhibiting their migration and tumor colony formation capabilities. These findings suggest that RVS not only acts as a direct inhibitor of MB-CSC growth but also functions as a radiosensitizer, potentially augmenting the effectiveness of radiotherapy for medulloblastoma. 

## 10. Proapoptotic and Antiproliferative Effects

Resveratrol demonstrates significant pro-apoptotic and anti-proliferative effects in various cancers. It induces apoptosis by increasing the expression of pro-apoptotic markers and tumor suppressor proteins like p53, as observed in cervical and breast cancer models [142,143]. In glioblastoma, it has been shown to suppress cell proliferation by modulating the PI3K/Akt and JAK/STAT3 pathways [17,144,145]. It disrupts cell cycle progression and reduces cancer cell viability in a dose-dependent manner [30,143,146]. 

A study by Hu et al. showed that resveratrol exerts both antiproliferative and proapoptotic effects on HBL-52 meningioma cells [147]. The antiproliferative impact is evident in the concentration- and time-dependent reduction in cell viability observed after treatment with resveratrol at varying concentrations (10–400 μM) and durations (24–48 h). Concurrently, resveratrol induces apoptosis, marked by increased cleaved caspase-3 levels and decreased pro-caspase-3 and Bcl-2 mRNA levels. The downregulation of Bcl-2, an anti-apoptotic protein, further underscores resveratrol’s pro-apoptotic influence. Crucially, these effects are linked to the upregulation of miR-34a-3p, a microRNA that targets Bcl-2 for downregulation. The confirmed binding of miR-34a-3p to the 3′ UTR (Untranslated Region) of Bcl-2 mRNA, coupled with decreased Bcl-2 protein levels upon miR-34a-3p overexpression, solidifies its role in mediating resveratrol’s effects. This dual action of inhibiting proliferation and promoting apoptosis positions RVS as a potential therapeutic agent for meningiomas.

Research by Ma et al. shows that resveratrol significantly inhibits MB (Medulloblastoma) cell proliferation, effectively slowing or halting cancer cell growth [148]. Furthermore, it induces apoptosis, promoting programmed cell death and eliminating harmful cancer cells. Interestingly, resveratrol’s mechanism of action involves down-regulating SIRT1 expression, a protein typically associated with cell survival. This contrasts with the expected SIRT1 activation and suggests that resveratrol’s pro-apoptotic effects are linked to SIRT1 inhibition. These findings highlight resveratrol’s potential as a safe and effective adjuvant treatment for medulloblastoma, possibly enhancing existing therapies and improving patient outcomes due to its accessibility from dietary sources. 

## 11. p53

The p53 protein, a critical tumor suppressor, plays a vital role in inhibiting cancer cell proliferation and promoting apoptosis in response to DNA damage [118,149,150]. Its mechanism of action involves halting the cell cycle at the G1/S transition, effectively preventing DNA replication under conditions of stress and genetic damage, thus safeguarding genomic integrity [151]. This regulatory function is particularly crucial in brain tumors, especially gliomas, where p53 mutations are frequently observed, contributing significantly to tumor progression [30,151]. Research by Clark et al. investigated the impact of resveratrol on p53 in glioma and glioblastoma stem-like cells [100]. Treating these cells with 100 µM of RSV led to increased expression of both p53 and its phosphorylated form, as demonstrated by Western blot analysis. Interestingly, this effect was calcium-dependent in glioma cells, showing reduced p53 levels in the presence of calcium, while GSCs (glioblastoma stem-like cells) did not exhibit this calcium dependency. Further supporting these findings, qPCR analysis revealed increased expression of p53-dependent downstream genes, including Bax, Pig8, and TP53INP, following RSV treatment. These results collectively suggest that RSV can modulate the expression and phosphorylation of p53, influencing its tumor-suppressing activity and potentially enhancing the activation of its downstream target genes involved in apoptosis and cell cycle regulation.

## 12. STAT 3 and JAK

Resveratrol holds promise as a supplemental therapy for gliomas, especially due to its interaction with the STAT3 (Signal Transducer and Activator of Transcription 3) signaling pathway. Overactive STAT3 is implicated in tumor growth and chemoresistance in glioblastoma [128,152]. Resveratrol effectively downregulates STAT3 activity [118,125,133,153], as demonstrated by Wu et al. [128]. Their study used qRT-PCR (Quantitative Reverse Transcription Polymerase Chain Reaction) to measure STAT3 mRNA levels in GBM cells treated with resveratrol, temozolomide, and a combination of both. After 48 h, STAT3 expression was significantly reduced in cells treated with either RSV or TMZ alone, with the combination treatment showing the greatest effect compared to TMZ alone. Immunocytochemistry staining corroborated these findings, revealing a reduced STAT3 signal, suggesting that RSV may enhance GBM cells’ response to TMZ. Furthermore, the study investigated RSV’s effect on STAT3’s negative regulators—PIAS3, SHP1, SHP2, and SOCS-3—using qRT-PCR. Results indicated that RSV, TMZ, and their combination upregulated PIAS3, SHP1, and SHP2, supporting a synergistic effect of RSV in boosting TMZ’s efficacy. While SOCS-3 expression did not show a clear trend, the overall data suggest that RSV improves GBM treatment responsiveness by reducing STAT3 activity and modulating its regulatory pathways.

Resveratrol, especially when combined with temozolomide, significantly inhibits STAT3 activation, reducing the expression of downstream effectors like Bcl-2 and survivin, which normally help cancer cells evade death [129]. This inhibition enhances glioma cell sensitivity to TMZ, making resveratrol a promising candidate for combination therapy in resistant GBM [128,129]. By targeting the STAT3 pathway, resveratrol not only suppresses glioma cell growth but also induces apoptosis and cell cycle arrest, potentially improving treatment outcomes in resistant GBM cases.

In addition to STAT3, resveratrol also modulates the JAK/STAT signaling axis, which is critical for glioblastoma pathogenesis. The Janus kinase family (JAK), consisting of JAK1, JAK2, JAK3, and TYK2, plays a critical role in regulating cytokine signaling pathways, influencing both immune cell functions and processes related to oncogenesis [154]. The JAK-STAT pathway is activated when a ligand binds to its corresponding receptor, triggering JAK kinase activation. This activation enables their kinase activity and leads to the phosphorylation of target proteins, notably STAT proteins [155]. These phosphorylated STAT proteins then form dimers, which translocate to the nucleus and bind to specific DNA sequences, initiating the expression of genes involved in cell proliferation, differentiation, and immune responses. Dysregulation of the JAK-STAT pathway, often caused by mutations, is implicated in various diseases [155], including cancers such as glioblastoma [156].

Zhang et al. investigated resveratrol’s impact on the inflammatory response within the GBM tumor microenvironment [157]. Their research demonstrated that resveratrol inhibits the JAK2/STAT3 pathway in GBM cell lines by decreasing JAK2 and STAT3 phosphorylation, as shown by Western blot analysis. Importantly, total protein levels of JAK2 and STAT3 remained unchanged, indicating that resveratrol modulates pathway activity rather than affecting kinase expression. Furthermore, immunofluorescence studies revealed reduced STAT3 translocation to the nucleus, where phosphorylated STAT3 typically activates genes associated with cell proliferation and survival. These findings suggest that resveratrol exerts a partial inhibitory effect on the JAK2/STAT3 pathway. By blocking JAK2, resveratrol can potentially reduce glioma cell proliferation and induce apoptosis [118,157]. This inhibition of JAK2 by RVS leads to the suppression of the signal transducer and activator of transcription 3 signaling [118]. As STAT3 is a transcription factor that promotes cell survival and proliferation when activated, inhibiting JAK2, and subsequently STAT3 allows resveratrol to exert growth-inhibitory effects on glioma cells [118,157]. Therefore, resveratrol’s ability to inhibit JAK2 and STAT3 signaling pathways positions it as a potential therapeutic agent in gliomas, leading to reduced tumor growth and increased apoptosis.

In glioblastoma, STAT3 activation is frequently linked to poor prognosis and chemoresistance [128,152]. Resveratrol has demonstrated the ability to inhibit tumor growth and enhance the sensitivity of cancer cells to chemotherapy by targeting the JAK-STAT signaling pathway, particularly through STAT3 downregulation [128,139]. This downregulation is significant because STAT3 activation is associated with GBM resistance to treatments like TMZ (temozolomide) [128]. Studies have shown that using a JAK2-specific inhibitor, AG490, resulted in reduced MGMT levels in LN428 cells mediated by STAT3 inactivation, suggesting that Res may influence MGMT through JAK-STAT modulation, thereby enhancing TMZ efficacy [128]. Combining RVS and TMZ not only reduces STAT3 activity but also increases the expression of negative regulators of STAT3, such as PIAS3, SHP1, SHP2, and SOCS3, further supporting Res’s inhibitory effect on the JAK-STAT pathway and its potential to improve chemosensitivity in GBM cells. Therefore, resveratrol’s ability to modulate JAK-STAT signaling, especially through STAT3 inactivation, highlights its potential as a therapeutic agent in combination with TMZ for treating glioblastoma, potentially overcoming chemoresistance and improving patient outcomes.

## 13. PI3K/Akt and AKT/PTEN

The PTEN/PI3K/Akt signaling pathway is crucial in cancer pathogenesis, regulating apoptosis, metabolism, proliferation, and cell growth [158]. PTEN, a phosphatase, dephosphorylates phosphatidylinositol-3,4,5-trisphosphate, a lipid product of PI3 kinase, into phosphatidylinositol [158]. This action reduces cellular PIP3 levels, a key signal for Akt kinase activation [158,159]. Consequently, PTEN acts as a negative regulator of the PI3K/Akt pathway [158,159], and mutations or loss of its tumor suppressor function are linked to the development of various cancers [158,159], including glioblastoma [160,161]. Dysregulation of the PTEN/PI3K/Akt pathway, often through PTEN loss, can lead to uncontrolled cell growth and survival, contributing to tumor development.

Resveratrol holds promise as an antitumor agent in glioblastoma multiforme, due to its ability to target the frequently dysregulated PI3K/Akt signaling pathway [118,144,145]. It downregulates the PI3K/Akt pathway, crucial for cancer cell proliferation and survival, by decreasing PI3K class III and phosphorylated Akt (p-Akt) expression, leading to reduced cell proliferation, invasion, and migration in glioblastoma cells [145]. RVS activation in gliomas contributes to tumor growth and therapeutic resistance [144,162,163]. Resveratrol effectively inhibits the PI3K/Akt pathway, resulting in decreased cell proliferation and increased apoptosis in glioma cells [100,144,145,162]. This targeted inhibition helps overcome resistance mechanisms that GBM cells develop against conventional treatments, which is crucial given their aggressive nature and ability to evade standard therapies [144,162,163,164]. In addition to PI3K/Akt pathway inhibition, resveratrol also promotes apoptotic cell death in glioma cells [144]. This dual action not only reduces tumor size but also enhances the effectiveness of other therapeutic agents when used in combination [144,162]. Resveratrol’s ability to modulate the PI3K/Akt pathway and promote apoptosis highlights its potential as a complementary treatment option for GBM, potentially improving outcomes and disease management.

Resveratrol inhibits the activation of the AKT signaling pathway, whose overactivation is often implicated in cancer progression [100,165]. By inhibiting AKT, resveratrol reduces glioblastoma cell survival [100,162,165]. Resveratrol may also enhance the activity of PTEN, a tumor suppressor that negatively regulates the AKT pathway, leading to decreased AKT signaling and contributing to tumor growth suppression and increased chemosensitivity [145,165,166]. This blockade of the AKT pathway by resveratrol not only inhibits glioblastoma cell proliferation but also plays a significant role in overcoming chemoresistance, a major challenge in glioblastoma treatment [162,163,165]. Resveratrol’s effects on the AKT/PTEN pathway involve complex molecular interactions, potentially modulating various upstream signals that influence PTEN activity and indirectly affecting AKT signaling [145,165]. Given its ability to target the AKT/PTEN pathway, resveratrol holds promise as a therapeutic agent in glioblastoma treatment, its dual action of inhibiting tumor growth and reversing chemoresistance making it a valuable candidate for further research and clinical application [118,162,165]. 

## 14. BAX and Bcl-2

Resveratrol modulates BAX and BCL-2, critical apoptosis regulators, impacting cancer cell survival. Mechanistically, RVS downregulates the ATF4/Chop/BCL-2/BAX signaling pathway, contributing to anti-aging and anti-cancer effects [167] and synergistic effects with agents like prednisolone enhance apoptosis through BAX and BCL-2 modulation [168].

Liu et al. investigated the combined effect of resveratrol and temozolomide on glioblastoma multiforme cells, examining Bcl-2 expression in rat and human (LN-18, LN-428) cell lines treated with or without RSV/TMZ (25/250 µM and 75/750 µM) for 48 h [129]. Western blot and immunocytochemistry revealed decreased Bcl-2 levels across all cell lines, with the most significant reduction (45.9%) in RG-2 cells treated with the lower RSV/TMZ dose compared to normal glioma cells. These findings suggest resveratrol’s potential in modulating apoptotic pathways by reducing Bcl-2 and inducing Bax, potentially enhancing glioblastoma and other cancer treatments.

In HBL-52 meningioma cells, resveratrol treatment significantly reduced Bcl-2 protein levels [147]. This decrease was linked to the upregulation of miR-34a-3p, a microRNA that directly targets Bcl-2. Western blot analysis confirmed lower Bcl-2 levels in cells transfected with the miR-34a-3p expression vector compared to controls. The observed reduction in Bcl-2, an anti-apoptotic protein, is significant as it promotes apoptosis, aligning with the increased apoptotic activity seen in resveratrol-treated HBL-52 cells. This suggests that resveratrol’s pro-apoptotic effect is partly mediated through Bcl-2 downregulation. Further confirming this mechanism, the study by Hu et al. demonstrated miR-34a-3p binding to the 3′ UTR of Bcl-2 mRNA, a crucial interaction for regulating Bcl-2 expression, validated through dual luciferase assays. In essence, resveratrol decreases Bcl-2 expression primarily via miR-34a-3p upregulation, promoting apoptosis and contributing to the compound’s antiproliferative effects.

## 15. NF-kB and Tumor Necrosis Factor

Nuclear factor kappa B (NF-κB) is a key transcriptional regulator responding to various stimuli [169]. Normally, NF-κB dimers are bound to inhibitory IκB proteins, preventing nuclear translocation [170]. Activation occurs in response to factors like pro-inflammatory cytokines, especially TNF-α [171], which activate the IκB kinase complex [170]. IKK phosphorylates IκB, leading to its degradation and releasing NF-κB to migrate to the nucleus [170]. There, NF-κB activates genes involved in angiogenesis, cell cycle regulation, apoptosis, tumorigenesis, and immune responses [171]. It is also central to pathways linking inflammation and carcinogenesis, and its dysregulation is implicated in various diseases, including glioma, contributing to pathogenesis and chemoresistance [169,172].

Resveratrol demonstrates anti-tumor effects in gliomas by significantly impacting NF-κB and TNF (tumor necrosis factor) signaling pathways. It inhibits glioma cell invasion and proliferation through various mechanisms. Resveratrol suppresses NF-κB activation by reducing IκB phosphorylation and nuclear p65 levels, decreasing NF-κB transcriptional activity [173]. It inhibits IκB kinase activity, preventing IκBα degradation, essential for NF-κB activation [174]. Additionally, RVS downregulates oncogenic microRNAs like miR-21, further inhibiting NF-κB activity [175]. Regarding TNF signaling, resveratrol reduces TNF-α-induced glioma cell invasion by inhibiting NF-κB activation and downregulating urokinase plasminogen activator (uPA) expression [176]. This mitigates the invasive properties of glioma cells often exacerbated by TNF signaling.

Jiao et al. [177] investigated the impact of resveratrol on glioblastoma-initiating cells, key drivers of GBM progression and recurrence, focusing on NF-κB p65 nuclear translocation. GICs (glioblastoma-initiating cells) were incubated with resveratrol at concentrations of 5 μM, 10 μM, and 20 μM for 48 h. Western blot analysis revealed that resveratrol inhibited NF-κB p65 nuclear translocation by increasing its cytosolic fraction and decreasing its nuclear fraction, confirmed by immunofluorescence staining. Furthermore, resveratrol reduced IKKα/β and IκBα phosphorylation without affecting IKKα/β expression levels. These findings suggest that resveratrol may inhibit glioma cell invasion by blocking NF-κB signaling pathway activation.

Huang et al. [178] examined the effects of temozolomide, resveratrol, and their combination on human glioblastoma multiforme cell strains. The cells were exposed to TMZ (100 μM), RSV (100 μM), or a combination of both for 24 h. The expression of κB-ras1, IκBα, and the NF-κB p65 subunit were then analyzed. TMZ exposure showed little effect on κB-ras1 and IκBα expression but significantly increased NF-κB p65 expression. Conversely, the TMZ + RSV combination minimally affected κB-ras1 and IκBα expression but significantly reduced NF-κB p65 expression. These results suggest that RSV may negatively regulate NF-κB activation, potentially by increasing IκBα expression, making the TMZ + RSV combination a possible strategy for overcoming TMZ resistance in GBM.

A study by Wen et al. investigated resveratrol’s impact on NF-κB signaling in medulloblastoma cells [153]. Resveratrol treatment initially activated NF-κB, increasing Bcl-2 expression. However, despite this initial pro-survival response, resveratrol ultimately induced apoptosis. Using PDTC (Pyrrolidine Dithiocarbamate), an NF-κB inhibitor, blocked NF-κB activation and Bcl-2 upregulation, accelerating resveratrol-induced apoptosis and bypassing neuronal differentiation. Conversely, LPS (lipopolysaccharide), an NF-κB activator, promoted sustained cell proliferation and differentiation. Tissue microarray analysis revealed significant differences in NF-κB activation (p65 nuclear translocation) between normal neurons and medulloblastoma tissues. These findings suggest a complex interplay between resveratrol and NF-κB in medulloblastoma, with initial survival pathway activation followed by cell death. NF-κB inhibition may enhance resveratrol’s anti-cancer effects, presenting a potential therapeutic strategy. 

Ryu et al. [176] studied the effects of TNF-α and resveratrol on human glioma cell invasion. Initially, glioma cells were exposed to varying TNF-α concentrations (1–20 ng/mL) for 24 h, and a Matrigel assay identified 10 ng/mL as the concentration inducing the highest invasion level. Subsequently, glioma cells were treated with 10 ng/mL TNF-α and varying RSV concentrations (5, 10, and 20 µM) for 24 h. Matrigel assays showed RSV inhibited TNF-α-induced invasion in a dose-dependent manner. MTT assays (colorimetric assay for assessing cell metabolic activity) revealed that neither RSV, TNF-α, nor their combination affected cell viability. Further investigation into TNF-α’s effect on NF-κB activation in glioma cells confirmed activation via Western blot detection of phosphorylated NF-κB. A total of 10 µM RSV reduced TNF-α-induced NF-κB phosphorylation by approximately 38%, 42%, and 58% after 1, 3, and 6 h of TNF-α stimulation, respectively. These results indicate that resveratrol can suppress glioma cell invasion by inhibiting TNF-α-induced NF-κB activation, highlighting its potential role in overcoming TNF-α-mediated chemoresistance.

## 16. VEGF

Resveratrol demonstrates significant anti-angiogenic properties, especially in gliomas [118,144,179], by inhibiting VEGF (Vascular Endothelial Growth Factor) signaling. Resveratrol directly binds to VEGF, disrupting its interaction with receptors, as identified through HerboChips screening [180]. This interaction inhibits several angiogenic processes in human umbilical vein endothelial cells (HUVECs). Resveratrol suppressed VEGF-induced endothelial cell proliferation (80 ± 9.01%), migration (140 ± 3.78%), invasion (110 ± 7.51%), and tube formation (120 ± 10.26%). Furthermore, resveratrol treatment decreased phosphorylation of VEGF Receptor 2, JNK (C-Jun-N-terminal-kinases), eNOS (endothelial nitric oxide synthase), Akt (Protein kinase B) and ERK (extracellular signal-regulated kinase). It also significantly reduced VEGF-induced ROS formation (50 ± 7.88% to 120 ± 14.82%). Thus, resveratrol inhibits VEGF-mediated angiogenesis through direct binding, altering signaling pathways, and reducing key angiogenic processes.

Research indicates that RVS reduces tumor growth and angiogenesis in glioma models through various mechanisms, including apoptosis induction and VEGF expression suppression [118,144,180]. Studies show that RVS treatment significantly lowers microvessel density (MVD) in glioma tissues, correlating with reduced tumor growth [118,181,182]. Furthermore, resveratrol promotes apoptosis in glioma cells, contributing to its anti-tumor effects [181,182].

In a study by Chang et al., involving 20 nude mice implanted with U87 human glioma cells, the mice were divided into four groups: two receiving resveratrol (10 mg/kg and 100 mg/kg), one receiving a vehicle treatment, and a blank control group [181]. The 100 mg/kg resveratrol group showed significant reductions in tumor volume and weight compared to the control groups (*p* < 0.05). Microvessel density, an indicator of angiogenesis, was significantly lower in the 100 mg/kg resveratrol group. Immunohistochemistry revealed a significant decrease in VEGF expression in the tumors of resveratrol-treated mice, correlating with the observed decrease in angiogenesis. A TUNEL assay showed a significant increase in apoptosis in tumor cells of mice treated with 100 mg/kg resveratrol (*p* < 0.05). These results demonstrate that resveratrol inhibits U87 glioma growth in nude mice by reducing tumor volume and weight, decreasing MVD and VEGF expression, and inducing apoptosis.

Tseng et al. found that resveratrol significantly inhibits angiogenesis in rat RT-2 gliomas, as evidenced by a marked reduction in microvessel density compared to control groups, particularly with 40 mg/kg/day (*p* < 0.002 and *p* < 0.01, respectively) [182]. The study also found resveratrol suppressed VEGF expression in RT-2 glioma cells, particularly at higher concentrations (10, 25, and 100 µM), reducing expression to 0.7, 0.5, and 0.2-fold of control levels, respectively. In vitro studies showed resveratrol inhibited ECV304 HUVEC proliferation in a concentration- and time-dependent manner, with an IC50 value indicating effective inhibition at various time points. Immunohistochemical analysis revealed a significant reduction in CD31 expression, further supporting resveratrol’s inhibition of glioma-induced angiogenesis.

Given its ability to inhibit angiogenesis and tumor growth, resveratrol may be a promising adjunct therapy for gliomas, potentially enhancing the efficacy of existing treatments.

## 17. Potential Aplication of Resveratrol Derivative—Pterostilbene

Pterostilbene (PTE), a natural dimethylated analog of resveratrol, has also demonstrated promising anti-glioma effects. Compared to resveratrol, pterostilbene has improved bioavailability and pharmacokinetic properties [183]. It has demonstrated promising anti-glioma effects in both in vitro and in vivo studies. PTE effectively inhibits the proliferation of various glioma cell lines (T98G, LN18, U87, LN229, and C6) in a dose- and time-dependent manner, inducing intrinsic mitochondria-mediated apoptosis as evidenced by increased levels of cleaved caspase-3, -9, and PARP-1, along with altered Bax, Bcl-2, and Survivin protein levels [184]. This apoptotic effect is mediated by a PTE-induced increase in reactive oxygen species production, as demonstrated by the reversal of PTE’s effects upon treatment with the antioxidant N-acetyl-l-cysteine. Furthermore, PTE causes S phase cell cycle arrest, marked by increased phospho-histone H2A.X and CHK2 protein levels, indicating DNA damage and checkpoint activation. PTE also inhibits glioma cell migration and invasion, reducing MMP-2 and MMP-9 expression. In vivo studies using a rat model have confirmed PTE’s efficacy, showing significant tumor volume reduction and prolonged survival without significant weight loss in treated animals.

Yu et al. showed that pterostilbene exhibits promising anti-glioma properties, as demonstrated by its multifaceted effects on U87 glioma cells [185]. The study showed significant inhibition of U87 cell proliferation after 48 h of treatment with pterostilbene (5 mM and 10 mM), compared to the control group (*p* < 0.05). Furthermore, pterostilbene effectively reduced the invasive potential of U87 cells, reinforcing its anti-tumor activity. Flow cytometry analysis revealed increased apoptosis in pterostilbene-treated U87 cells, indicating the compound’s ability to promote programmed cell death. This pro-apoptotic effect was further supported by dose-dependent changes in Bcl-2 and Bax expression: pterostilbene decreased Bcl-2 mRNA and protein levels while increasing Bax levels (*p* < 0.05). The collective findings underscore pterostilbene’s promising potential as a therapeutic agent for glioma treatment. PTE has demonstrated the ability to inhibit glioma cell proliferation and invasion, while concurrently inducing apoptosis, highlighting its multifaceted anti-tumor effects.

A study by Chen et al., investigating pterostilbene’s anti-glioma effects, revealed its multi-faceted impact on glioma cells [186]. Network pharmacology analysis identified 37 anti-glioma targets of PTE, enabling the construction of target and protein-protein interaction networks that predicted PTE’s potential mechanisms of action. CCK-8 assays demonstrated PTE’s significant reduction in U87MG and GL261 glioma cell viability and proliferation in a concentration-dependent manner. Furthermore, PTE inhibited glioma cell migration and adhesion, as observed through clone formation and cell scratching assays. PTE also induced apoptosis, evidenced by morphological changes visualized with Hoechst staining and decreased mitochondrial membrane potential in U87MG cells. Concurrently, PTE triggered pyroptosis, marked by increased LDH (lactate dehydrogenase) release and characteristic morphological features observed under microscopy, along with a rise in propidium iodide-positive cells indicating membrane integrity compromise. Western blot analysis confirmed these findings, revealing PTE-induced upregulation of apoptosis-related proteins (cleaved PARP1 and BAX) and downregulation of Bcl-2, alongside increased activation of pyroptosis-related proteins (cleaved caspase-3 and GSDME-N). These results collectively demonstrate PTE’s potent anti-glioma activity through the activation of both caspase-3/GSDME-mediated pyroptosis and mitochondrial apoptosis pathways, in addition to inhibiting cell viability, proliferation, and migration.

Research by Schmidt et al. indicates that pterostilbene when combined with gefitinib and sertraline, exhibits promising effects against glioblastoma [187]. This combination therapy suppressed cell growth and viability across a panel of 41 patient-derived glioblastoma cell cultures, suggesting broad applicability. Moreover, the combination significantly inhibited malignant phenotypes such as sphere formation and migration. Interestingly, the potentiating effect of pterostilbene varied among different glioblastoma cultures, correlating with specific genetic mutations like EGFR (Epidermal Growth Factor Receptor) and PIK3CA missense mutations and 1p32 focal deletions, suggesting potential personalized treatment strategies. Pterostilbene’s mechanisms of action include inducing cell cycle arrest, enhancing MAPK (mitogen-activated protein kinase) signaling inhibition when combined with gefitinib, and inducing TXNIP (thioredoxin-interacting protein) expression, leading to increased oxidative stress and apoptosis. Furthermore, in vivo studies are needed to validate these findings and explore the underlying mechanisms in greater detail, but the results suggest pterostilbene may be a valuable nontoxic addition to existing glioblastoma treatments, especially for genetically defined patient subsets.

Huynh et al. highlight the potential of PTE in targeting glioma stem cells (GSC), which contribute to treatment resistance and tumor recurrence in glioblastoma multiforme [188]. The study linked the high expression of GRP78, a stress-associated protein, to enhanced GSC characteristics such as migration, invasion, and self-renewal, particularly in CD133-positive GSCs. Silencing GRP78 suppressed these GSC properties and increased sensitivity to irradiation, suggesting GRP78 as a potential therapeutic target. The study also identified a negative correlation between GRP78 and miR-205 levels, with miR-205 induction suppressing GRP78 and modulating GSC characteristics and irradiation resistance. Pterostilbene treatment significantly suppressed GSC self-renewal and irradiation resistance by increasing miR-205 levels and negatively modulating GRP78 signaling. Furthermore, PTE suppressed tumorigenesis in GSC xenograft mouse models. These statistically significant results (using ANOVA and post hoc comparisons) suggest that targeting the GRP78/miR-205 axis with pterostilbene may be a promising strategy to overcome irradiation resistance in GBM.

## 18. Conclusions

Resveratrol demonstrates significant therapeutic potential due to its modulation of various signaling pathways and cellular processes. Its low toxicity and ability to target multiple molecular signaling pathways make it a promising antineoplastic agent, particularly against central nervous system tumors. Resveratrol crosses the blood–brain barrier, suppressing oxidative stress and inflammation, inhibiting cell proliferation, and triggering cell death mechanisms. It modulates various signaling pathways, including NF-κB, TNF, p53, Wnt, PI3K/AKT/mTOR, AKT/PTEN, STAT3, JAK, BAX, Bcl-2, and VEGF, and can alleviate resistance to temozolomide or enhance radiotherapy’s efficacy. However, further research is needed to address its poor bioavailability and define its efficacy in different brain cancer subtypes. Optimizing resveratrol’s delivery systems, formulations, and developing analogs could enhance its anticancer activity and clinical use.

Reviewed literature also highlights the multifaceted anti-tumor effects of the resveratrol derivative—pterostilbene—in glioma and glioblastoma models. It demonstrates promising potential in protecting against CNS (central nervous system) disorders through various mechanisms. It exhibits antioxidant activity, reducing oxidative stress; anti-inflammatory effects, mitigating inflammation; regulation of lipid metabolism, crucial for neuronal health; and improvement of synaptic function, enhancing neurogenesis and cognitive function. Furthermore, pterostilbene inhibits glioma progression by inducing cell cycle arrest and inhibiting migration and invasion. These effects are mediated through modulation of several key molecular pathways, including AMPK/STAT3, Akt, NF-κB, MAPK, and ERK signaling. While current evidence suggests pterostilbene’s safety, further research is needed to fully assess its pharmacokinetics and safety profile in clinical settings. More comprehensive studies, including both animal models and human trials, are crucial to validate pterostilbene’s therapeutic applications and deepen our understanding of its effects and mechanisms in CNS disorders.

## Data Availability

No new data were created or analyzed in this study. Data sharing is not applicable to this article.
